# Effects of transcranial LED therapy on the cognitive rehabilitation for diffuse axonal injury due to severe acute traumatic brain injury: study protocol for a randomized controlled trial

**DOI:** 10.1186/s13063-018-2632-5

**Published:** 2018-04-24

**Authors:** João Gustavo Rocha Peixoto dos Santos, Ana Luiza Costa Zaninotto, Renato Amaro Zângaro, Ana Maria Costa Carneiro, Iuri Santana Neville, Almir Ferreira de Andrade, Manoel Jacobsen Teixeira, Wellingson Silva Paiva

**Affiliations:** 1 Department of Neurological Surgery, University of São Paulo School of Medicine, 255 Dr. Enéas de Carvalho Aguiar Av., São Paulo, SP 05403-010 Brazil; 2 Division of Neuropsychology, University of São Paulo General Hospital, São Paulo, Brazil; 3 Center for Innovation, Technology and Education (CITÉ) SJ dos Campos, São Paulo, 12245-650 Brazil; 4 University of São Paulo School of Nursing, São Paulo, Brazil

**Keywords:** Brain injuries, Diffuse axonal injury, Low-level light therapy, Neurologic manifestations, Quality of life, Brain diseases, Trauma, Central nervous system diseases, Nervous system diseases, Craniocerebral trauma

## Abstract

**Background:**

Photobiomodulation describes the use of red or near-infrared light to stimulate or regenerate tissue. It was discovered that near-infrared wavelengths (800–900 nm) and red (600 nm) light-emitting diodes (LED) are able to penetrate through the scalp and skull and have the potential to improve the subnormal cellular activity of compromised brain tissue. Different experimental and clinical studies were performed to test LED therapy for traumatic brain injury (TBI) with promising results. One of the proposals of this present study is to develop different approaches to maximize the positive effects of this therapy and improve the quality of life of TBI patients.

**Methods/design:**

This is a double-blinded, randomized, controlled trial of patients with diffuse axonal injury (DAI) due to a severe TBI in an acute stage (less than 8 h). Thirty two patients will be randomized to active coil helmet and inactive coil (sham) groups in a 1:1 ratio. The protocol includes 18 sessions of transcranial LED stimulation (627 nm, 70 mW/cm^2^, 10 J/cm^2^) at four points of the frontal and parietal regions for 30 s each, totaling 120 s, three times per week for 6 weeks, lasting 30 min. Patients will be evaluated with the Glasgow Outcome Scale Extended (GOSE) before stimulation and 1, 3, and 6 months after the first stimulation. The study hypotheses are as follows: (1) transcranial LED therapy (TCLT) will improve the cognitive function of DAI patients and (2) TCLT will promote beneficial hemodynamic changes in cerebral circulation.

**Discussion:**

This study evaluates early and delayed effects of TCLT on the cognitive rehabilitation for DAI following severe acute TBI. There is a paucity of studies regarding the use of this therapy for cognitive improvement in TBI. There are some experimental studies and case series presenting interesting results for TBI cognitive improvement but no clinical trials.

**Trial registration:**

ClinicalTrials.gov, NCT03281759. Registered on 13 September 2017.

**Electronic supplementary material:**

The online version of this article (10.1186/s13063-018-2632-5) contains supplementary material, which is available to authorized users.

## Background

Annually, there are 50,000 deaths from traumatic brain injury (TBI) in the USA, and there are 235,000 hospitalizations from non-fatal TBI. Recently, there has been an increase of 14% of TBI cases presenting to the emergency department [1, 2].

Recent studies show that about 124,000 (43.1%) of TBI patients discharged from acute hospitalizations developed TBI-related long-term disability [3]. The definition of disability was focused on findings from a previous population-based study, which included considerable difficulty or inability to perform activities of daily living, the presence of a post-injury symptom preventing patients from doing things they intend to do, and poor cognitive and mental health levels on standard measures [4].

It is relevant to mention that these data are likely underestimated, as the data published are solely from hospitalizations only. Thus, they probably do not include TBI patients treated in other settings or for whom treatment was not sought [3].

### Radiological features of DAI and clinical presentation

Recent data accepted that diffuse axonal injury (DAI) is now one of the most common types of primary lesions in patients with severe head trauma [5]. DAI not accompanied by an intracranial mass lesion occurs in approximately 50% of patients with severe TBI and causes 35% of all TBI deaths [6].

Patients with DAI present with important impairment of consciousness from the moment of trauma [7]. DAI is the most common cause of persistent vegetative state and severe disability in TBI [8]. Damage to the reticular activating system is proportional to the level and duration of coma noted after DAI [6]. Autonomic dysfunction symptoms seen in DAI (such as temperature control and satiety center disorders) have been associated with injury to the hypothalamus, which controls these activities [6].

In the chronic phase, DAI in milder forms can lead to residual neuropsychiatric problems and cognitive deficits, including concentration difficulties, focal neurologic lesions, memory loss, decreased attention span, intellectual decline, psychiatric disturbances, headaches, and seizures [5]. These disability symptoms are known as post-concussion syndrome [9]. DAI should be suspected in all closed head injury patients with persistent and significant impairment of consciousness in the acute phase or residual neuropsychiatric deficits in the chronic phase [5].

Disability symptoms caused by brain lesions are not usually evident in structural computed tomography (CT) or magnetic resonance imaging (MRI) [10–13]. CT may show some petechial foci of hemorrhage in the acute phase, but it underestimates DAI extent because less than 20% of DAI lesions are hemorrhagic [5]. Other radiologic features which could be appear are as follows: (1) single or multiple small intraparenchymal hemorrhages in the cerebral hemispheres (<2 cm in diameter); (2) intraventricular hemorrhage; (3) hemorrhage in the corpus callosum; (4) small focal areas of hemorrhage adjacent to the third ventricle (<2 cm in diameter); (5) brain stem hemorrhage [14]. Intraventricular hemorrhage arises from disruption of the subependymal plexus of the capillaries and veins that lie along the ventricular surface of the septum pellucidum, fornix, and corpus callosum [15].

DAI is caused by shearing forces which transect small blood vessels running in parallel with axons, leading to detectable microscopic hemorrhages. Gradient echo and T2*-weighted MR images detect signal dropout provoked by ion-containing heme groups in slow-moving blood [16]. MRI is more sensitive in the identification of shearing injuries because it enables detection of non-hemorrhagic as well as hemorrhagic lesions. The axoplasmic leakage and edema around areas of neuronal disruption in non-hemorrhagic lesions are more conspicuous on T2-weighted images [5]. Diffusion tensor imaging (DTI) MRI scans can show DAI signs [12, 17]. DTI is sensitive to detect water diffusion in the brain, presenting microstructural lesions in white matter tracts [18].

There are five white matter regions of interest where one can usually see the lesions:

1. Anterior corona radiata (41% of TBI cases)
2. Uncinate fasciculus (29% of TBI cases)
3. Genu of the corpus callosum (21% of TBI cases)
4. Inferior longitudinal fasciculus (21% of TBI cases)
5. Cingulum bundle (18% of TBI cases) [19]

The loss of connectivity in the aforementioned tracts (DAI) is responsible for all the symptoms and signs of disability following TBI [11].

### Principles of transcranial red/NIR LED photobiomodulation

The use of red or near-infrared (NIR) light to stimulate or regenerate tissue is called photobiomodulation. Trying to repeat an experiment performed in Boston, USA, the Hungarian Endre Mester accidentally discovered this process. He tried to use laser beams to destroy cancer cells experimentally implanted into a laboratory rat [20]. His mistake was to build a ruby laser that was only a tiny fraction of the power of the laser that had previously been used in Boston by McGuff [21]. Thus, he noticed that hair had regrown and wound had healed in the stimulated area where the tumor had been implanted [20].

Sometime after that, about 30 years ago, cadaver studies showed that NIR wavelengths (800–900 nm) and red (600 nm) could penetrate through the scalp and skull (about 2–3% of them can penetrate 1 cm from the cortex) [22, 23]. They also have the potential to improve the subnormal cellular activity of compromised brain tissue.

Photobiomodulation is a therapeutic tool for hypoxic, infected, and ischemic wounds. It also shows effectiveness in healing, reducing edema and inflammation, relieving pain, treating chronic inflammation and autoimmune diseases, recovery from ischemic heart disease, and attenuating degeneration of the optic nerve [24, 25]. Multiple studies testing its effects in TBI are in course [10, 20, 26, 27].

Wavelengths within the far-red to the near-infrared range (630–1000 nm) along with a minimal energy density of 4 J/cm^2^ are the optimal therapeutic intervention [28]. Stimulating biological processes proved to be effective with these wavelengths and energy density. It is believed that they act by four main actions:

1. Stimulating mitochondria to increase adenosine triphosphate (ATP) production [29, 30] through increased cytochrome c oxidase (CCO) effect. CCO acts by absorbing light photon and avoiding nitric oxide (NO) inhibitory effect on itself. Thus, there is an increase in mitochondrial membrane potential, providing more oxygen consumption, as soon as more glucose is metabolized, and more ATP is produced [29].
2. Inhibition of microglial activation and also acting as a strong antioxidant promoting an anti-inflammatory effect (increase in mitochondrial superoxide dismutase) [31, 32].
3. Increasing regional cerebral blood flow [26, 33].
4. Changing protein expression through signaling mediators and activation of transcription factors. These changes last for some considerable time and explain the long-lasting effect [21].
5. Promoting *neurogenesis* and *synaptogenesis*. However, this was only seen in studies with small animals treated with NIR in the acute stage post-TBI [27, 32, 34].

Neurogenesis is the denomination given to the process of differentiation of a progenitor cell into a neuron. The subventricular zone and hippocampus are the areas where this process usually occurs. Another interesting fact is that it is not such a rare event in the brain as we once thought [35]. Synaptogenesis is a process of formation of new synapses. It is one of the brain recovery mechanisms after TBI [36].

Current small studies with acute severe TBI mice used transcranial NIR photobiomodulation to treat them. These studies have supported the notion that transcranial photobiomodulation increases neurogenesis and synaptogenesis. The protocol began 4 h post-TBI, and they discovered that there was significantly better recovery at 28 days post-TBI in mice receiving three daily transcranial NIR photobiomodulation treatments when compared to controls. The neurogenesis was notedly increased when measured by immunofluorescence staining of brain sections [37, 38].

The estimated mean of NIR light penetration depth through the scalp and skull is 23.6 ± 0.7 mm [39–41]. Considering the different areas of the skull, the percentage of penetration also changes (0.9% at the temporal region, 2.1% at the frontal region, and 11.7% at the occipital region) [42]. Transcranial photobiomodulation application is easier in the forehead, because of the absence of hair and longer wavelength, which facilitates NIR light penetration [20].

### Study purpose and objectives

The purposes of this study are to evaluate early and delayed effects of transcranial light-emitting diode (LED) therapy (TCLT) and determine whether this therapy is effective for the cognitive rehabilitation of DAI patients after TBI.

### Primary outcome measures

The primary hypothesis is that there will be evidence of improvement in functional outcome measured by the Glasgow Outcome Scale Extended (GOSE) after stimulation in comparison to the placebo group at 3 months.

### Secondary outcome measures

Improvement effects of TCLT in follow-up images will be measured by the Marshall computed tomography scale and Adams grading scale for DAI. The hemodynamic improvement effect of TCLT will be measured by transcranial Doppler (systolic and diastolic velocities of the left middle cerebral and basilar arteries, and pulsatility index and resistance index values).

## Methods/design

### Study design

This study is a sham-controlled, randomized, single-center trial where data will be collected from patients admitted in the emergency service of University of São Paulo General Hospital, São Paulo, Brazil. The recruitment period started in October 2017, and the estimated data collection conclusion is October 2018.

**Fig. 1:**
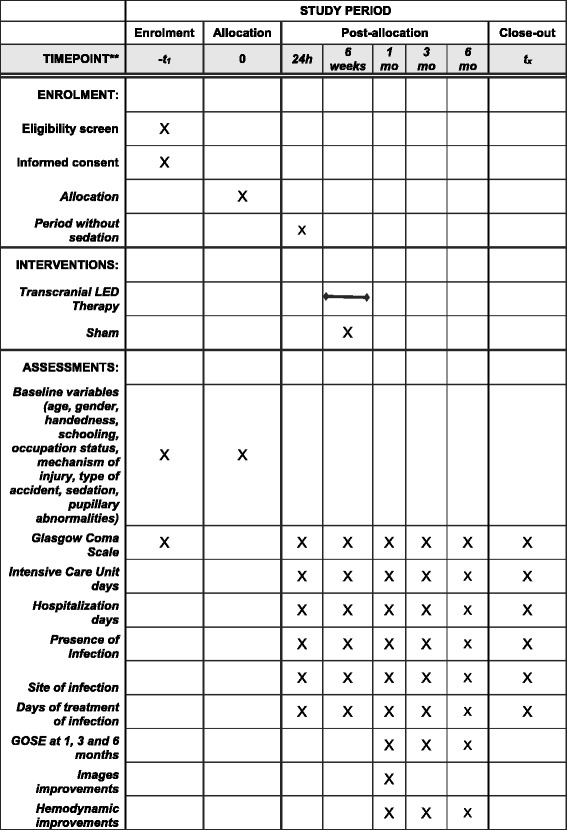
Template of content for the schedule of enrollment, interventions, and assessments from the LED TBI protocol. Baseline variables are evaluated before the intervention. The primary variable is evaluated 3 months after the intervention, and the secondary variables are evaluated during the whole period of this study. *GOSE* Glasgow Outcome Scale Extended

The present trial will respect CONSORT (Consolidated Standards of Reporting Trials) guidelines and SPIRIT (Standard Protocol Items: Recommendations for Interventional Trials) guidelines as shown in Fig. 1 and Additional file 1, respectively.

### Sampling

The eligibility criteria will include adult patients between 18 and 60 years who are victims of a severe (Glasgow Coma Scale ≤8) acute (hospital admission within less than 8 h from trauma) TBI and present with the DAI diagnosis. Patients younger than 18 years old will be excluded due to the known different physiopathology typical of this age. We excluded patients older than 60 because different studies revealed that these patients had a significantly worse outcome after TBI. Six months after severe head injury, 92% were dead, vegetative, or severely disabled. Four class I studies demonstrated a mortality of >75% in severely brain-injured patients older than 60. The critical age threshold for worsening prognosis appears to be above 60 in a review of class I and II studies [43].

DAI diagnosis is histopathological. It can be clinically defined by coma lasting 6 h or more after TBI, excluding cases of swelling or ischemic brain lesions [44, 45]. Focal brain injury may produce mass effects from hemorrhagic contusion or hematoma, which can induce herniation and brain stem compression, and the resultant may be coma, which is not usually immediate but develops in a secondary fashion. Otherwise, DAI can be a sole source of post-traumatic coma in the absence of mass lesions [45]. MRI will be performed in all patients for documentation. However, MRI cannot demonstrate DAI lesions in up to 10% of patients. Therefore, patients with Glasgow Coma Scale (GCS) ≤8 lasting more than 6 h without MRI or with normal CT or normal MRI will also be diagnosed as having DAI, if no other reasons justify the coma status [44–46]. Patients will be considered to have awakened from coma when they score 6 points in the best motor response in the GCS, consequently not being included in this protocol.

In summary, the inclusion criteria will be:

1. Coma lasting more than 6 h without sedation
2. CT scan without focal surgical lesions (Marshall I and II) and no signs of intracranial hypertension
3. Transcranial Doppler and optic nerve sheath US with no signs of intracranial hypertension
4. Admission within less than 8 h of trauma

The exclusion criteria will be:

1. History of drug or narcotic abuse
2. Emergence of surgical lesions or signs of intracranial hypertension in follow-up CTs
3. Transcranial Doppler or optic nerve sheath US presenting signs of intracranial hypertension
4. Psychiatric disorders
5. Injury severity score ≥3, according to the Abbreviated Injury Scale [47]
6. Bilateral fixed and dilated pupils

The optic nerve sheath is contiguous with the subarachnoid space. Thus, an increase in ICP results in a corresponding increase in the optic nerve sheath diameter. The cutoff point which will be considered intracranial hypertension in this protocol is ≥5 mm [48].

The transcranial Doppler can estimate ICP through a formula, calculated using the mean systemic arterial blood pressure (ABP), the arterial resistivity index (RI), and the mean flow velocity of the cerebral arteries (MFV): ABP × RI/MFV [49].

### Severe TBI evaluation and management at University of São Paulo General Hospital

At University of São Paulo General Hospital, all severe TBI patients are managed in an intensive care unit. An ICP monitoring catheter is inserted in patients who present with the criteria for ICP monitoring [50]:

GCS ≤8 and:

- ▪ Marshall ≥2 head CT scan or
- ▪ Normal head CT scan and at least two of the following three criteria:
  - PAS ≤90 mmHg
  - Age >40
  - Pathological posture (decerebration or decortication)

The decision for catheter insertion is made by the on-call attending neurosurgeon. Patients in whom the catheter cannot be inserted for any reason are evaluated by non-invasive strategies (optic nerve sheath ultrasonography or transcranial Doppler) for ICP monitoring and CT scan. In our center, all TBI patients are submitted to head CT scans at admission, within 6 h after trauma, on the third and the fifth day after trauma, or before these times if they present any neurological deterioration (which is commonly defined as a decrease of two or more points in the motor component of the Glasgow Coma Scale) [51]. When patients need to be hospitalized for longer than 5 days, the decision for the following head CT scans is individualized. All TBI patients in coma are evaluated with electroencephalogram (EEG) for seizure diagnosis in the intensive care unit (ICU).

### Recruitment

A total of 32 patients, of both genders, from 18 to 60 years old who meet the inclusion and exclusion criteria will be evaluated. All eligible recruited patients will be admitted to the Emergency Department of University of São Paulo General Hospital and randomly divided into two groups (active coil LED helmet vs sham). All patients will receive a unique identification number, which will be used along the study to keep the staff blinded.

### Randomization and blinding

The randomization process will be achieved through a computer software, respecting a 1:1 ratio for the 30 patients. The method of randomization used by the computer software is random permuted block sizes of 2. The two groups will be active coil and sham coil. As this is a double-blinded study, neuropsychologists and patients (as well as their relatives) will be blinded to the group assignment. The method of blinding will consist of using an equal helmet for both the intervention and placebo groups. Both helmets will emit a red light, but only the active coil group will receive LED emission (intervention group).

### TCLT protocol

Participants will be submitted to TCLT at a 627-nm wavelength, with 0.2-W LED power, 70-mW/cm^2^ power density, 10-J/cm^2^ energy density, 13-mm spot size, and 40 J/cm^2^ per treatment. These parameters were chosen based on the optimal wavelength penetration within biological tissues (wavelength range of 600–1000 nm). At short wavelengths, absorption occurs predominantly by chromophores such as melanin and hemoglobin.

**Fig. 2:**
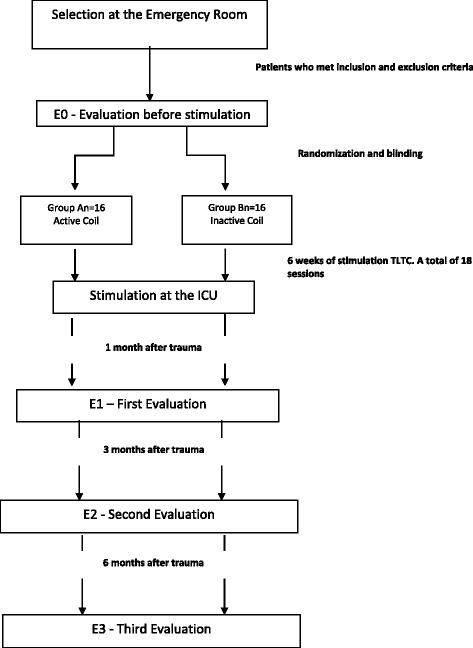
Study protocol flow diagram. Thirty patients who meet the inclusion and exclusion criteria will be recruited from the Emergency Department of University of São Paulo General Hospital. After baseline data collection, GOSE evaluation, transcranial Doppler hemodynamic measures, and CT scan, these patients will be randomized at a 1:1 ratio: active coil group vs sham coil group. They will be submitted to 18 sessions of TCLT three times per week and reevaluated with the aforementioned instruments after 1, 3, and 6 months after trauma

In order to avoid skin burning, the power density of 70 mW/cm^2^ was considered safe. The wavelength (approx. 627 nm) is characterized by a relatively low energy density (40 J/cm^2^) for therapeutic uses. The application of LED irradiation will be in four points in the frontal and parietal regions for 30 s at each point (120 s total/session) three times per week during 6 weeks. The flow chart in Fig. 2 explains the protocol steps.

During the TCLT sessions, patients will remain seated in bed with head support and with their eyes protected. Eighteen sessions will be carried out, with a total of 56 applications. The same qualified previously trained therapist will perform all TCLT sessions at the same time of the day.

### Tolerability and safety

All patients will be evaluated after each session. If a major side effect is noticed, medical assistance will be provided for the patient and further examination will be done as necessary. This fact will be written down and discussed to avoid future occurrences. Depending on the case, exclusion of the patient will be considered.

There are no studies with photobiomodulation (PBM) for severe TBI patients in the acute stage of trauma. Considering that fact, some safety precautions will be taken. There are no papers studying the possible effect of increasing the ICP due to PBM. But some chronic-stage studies [52] noticed a blood flow increase. The possible ICP increase due to that will be monitored by ICP catheter insertion or non-invasive methods (when catheter insertion cannot be performed for any reason). Because of the improvement effect in chronic TBI patients, the authors do not believe that this intervention could be harmful. ICP monitoring will add safety to this study. If elevation of ICP happens, the authors will stop the session and observe if intervention for ICP control is required. The patient will be evaluated in the following session, and if elevation happens again, the patient will be excluded.

Another concern would be a possible deleterious effect produced by the heat generated by the helmet in acute-stage TBI patients. Some studies showed an insignificant cortical temperature increase [52, 53]. Our device has already been considered safe in this regard; in addition, the protocol includes monitoring the head skin and global temperature of the patient during the sessions. If there is harmful elevation of skin temperature in the area of stimulation, the session will be interrupted and the patient will be reevaluated. If this effect is noticed in the following session, exclusion of the patient will be considered for safety.

Low-level light therapy (photobiomodulation), which is the principle of therapy of this protocol, is registered on the Food and Drug Administration, and the adverse effects described are ocular injuries and electrical shock [54]. Ocular injuries should not happen in this protocol since the LED will not be applied close to the eyes. But, for safety, the eyes will be evaluated for redness or irritation after each session. Electrical shock is unlikely in this protocol because the helmets are supplied by internal batteries. However, it will be reported and dealt with by the engineer responsible for maintenance of the helmet, if it happens.

### Instruments of evaluation

#### Glasgow Outcome Scale Extended

It is an expansion [55] with the objective of complementing the limitation of the original Glasgow Outcome Scale [56]. Despite being more practical, the five original groups (Dead, Vegetative State, Severe Disability, Moderate Disability, Good Recovery) of the original scale are broad and therefore insensitive to subtle changes in functional status [55]. The 8-point Glasgow Outcome Scale Extended (GOSE), developed only in 1981, provides different criteria to subdivide the upper three categories of the scale: Death, Vegetative State, Lower Severe Disability, Upper Severe Disability, Lower Moderate Disability, Upper Moderate Disability, Lower Good Recovery, Upper Good Recovery [57].

#### Adams grading scale for diffuse axonal injury

In 1989, this classification was first published grading diffuse axonal injury [58]:

- Grade 1: histological evidence of axonal injury in the white matter of the cerebral hemispheres, the corpus callosum, the brain stem, and, less usually, the cerebellum.
- Grade 2: there is also a focal lesion in the corpus callosum.
- Grade 3: there is, in addition, a focal lesion in the dorsolateral quadrant or quadrants of the rostral brain stem.

In the present study, the principles of the histological Adams grading scale will be applied on the MRI scans of the patients.

#### Marshall computed tomographic classification

The first Marshall classification based on computed tomographic abnormalities was published in 1991 [59] and was composed of four groups of patients. In 2005, these criteria were reviewed and two new groups were added [60]. Recently, the Marshall computed tomographic classification has six different groups:

- Diffuse injury I (no visible pathology)—no visible intracranial pathology seen on the CT scan.
- Diffuse injury II—cisterns are present with a midline shift of 0–5 mm and/or lesion densities present; no high- or mixed-density lesion 25 cm^3^; may include bone fragments and foreign bodies.
- Diffuse injury III (swelling)—cisterns compressed or absent with a midline shift of 0–5 mm; no high- or mixed-density lesion 25 mm.
- Diffuse injury IV (shift)—a midline shift of 5 mm; no high- or mixed-density lesion 25 cm^3^.
- Evacuated mass lesion V—any lesion surgically evacuated.
- Non-evacuated mass lesion VI—high- or mixed-density lesion 25 cm^3^; not surgically evacuated.

#### Gennarelli’s clinical classification

This is a classification of the severity of DAI with three levels: in mild DAI, coma lasts 6–24 h. In moderate DAI, coma lasts longer than 24 h but there is no abnormal posturing. In severe cases of DAI, the coma duration is longer than 24 h and signs of brain stem impairment (decortication or decerebration) can also be found [44].

#### Abbreviated Injury Scale

It is an anatomically based scale created in a global consensus for scoring the severity of trauma. It is a 6-point ordinal scale: (1) minor, (2) moderate, (3) serious, (4) severe, (5) critical, (6) maximal (currently untreatable) [47].

### Cerebral blood flow evaluation

#### Transcranial Doppler

All patients will be evaluated by transcranial Doppler before the first stimulation and after the last one. Evaluation will be done by a doctor expert in transcranial Doppler who will not be aware of the patient’s group (intervention vs sham). The transcranial Doppler parameters of evaluation include the following: blood flow velocities (systolic and diastolic) of the bilateral middle cerebral arteries as well as the basilar artery. The device that will be used for measuring is a transcranial Doppler ultrasound (LOGIQ; GE Healthcare Systems, Wauwatosa, WI, USA, 2012) with a 2-MHz transducer. The mean flow velocity (cm/s) will be obtained every second (sampling rate of 1 Hz) and saved for analysis. The three best measures of the systolic velocity, diastolic velocity, pulsatility index, and resistance index will be selected to calculate the mean values [52].

Evaluation with the transcranial Doppler will be performed by one expert doctor only to maintain a uniform measurement of TBI patients.

### Outcome variables

#### Primary

- Improvement in functional outcome measured by the GOSE after 3 months of the first stimulation [57].

The authors decided for an approach based on dichotomizing the GOSE (favorable vs unfavorable response). For analysis of outcomes at 3 months, the authors pre-specified death, persistent vegetative state, and severe disability on the GOSE as unfavorable outcomes. Analyzing the GOSE on a binary outcome measure reflects success or failure more plainly, which simplifies the matter of assigning a score to a patient too cognitively impaired to be tested. Moreover, describing the study on this way eases the interpretation for clinical significance [61]. Many studies in TBI [61–63] analyze and prefer to interpret this scale in its dichotomized form.

#### Secondary

- Improvement in early and delayed functional outcomes measured by the GOSE after 1 and 6 months of the first stimulation.
- Improvement effects of TCLT in follow-up images measured by the Marshall computed tomography scale [60], the Adams grading scale [58], and Gennarelli’s clinical severity scale [44] for diffuse axonal injury.
- Hemodynamic improvement effect of TCLT measured by transcranial Doppler (systolic and diastolic velocities of the middle cerebral (MCA) and basilar arteries (BA) and the pulsatility index and resistance index values) [44]. It will be evaluated before stimulation and 1, 3, and 6 months after the first stimulation.

For GOSE 1 and 6 months after the first stimulation, a similar calculation of the primary variable outcome will be used.

Patients will repeat the CT scan and the MRI after 1 month of treatment, and they will be evaluated again for the following scales: Gennarelli’s, Adams’, and Marshall’s, and a comparison with image findings of the sham group will be performed.

The transcranial Doppler will analyze systolic and diastolic velocities of the MCAs and BAs and pulsatility index and resistance index values. The means and standard deviation will be calculated for these variables and compared with the first evaluation before stimulation.

### Baseline measures

Complementary data will be assessed from all the patients:

- Sociodemographic characteristics: age (years), gender (male/female), handedness (right/left/ambidextrous), level of schooling (none, preschool, elementary school, middle school, high school, tertiary or higher school), occupation status
- Characteristics related to the trauma: mechanism of injury, type of accident
- Characteristics at admission: sedation (no/yes), pupillary abnormalities (both pupils reactive, one reactive, and none reactive to light), Glasgow Coma Scale
- Characteristics during hospital stay: ICU days, hospitalization days, infection during hospitalization (no/yes, site of infection, days of treatment), Glasgow Coma Scale at the trauma scene, at admission, at discharge from the ICU (or death), at discharge from hospital (or death)
- Presence of dysautonomia related to DAI

The mechanisms of injury which will be considered are as follows: blow to the head (direct impact), acceleration-deceleration forces (wherein no direct impact is required), and concussive forces (blast waves from explosions).

The types of accident are as follows: motor vehicle collisions, motorcycle crashes, pedestrians struck by automobiles, falls, explosions.

Dysautonomia is a syndrome characterized by episodes of autonomic dysregulation, leading to an increased heart rate, respiratory rate, and body temperature and other symptoms. It can be defined by simultaneous occurrence of five of the following features [49]:

1. Increased heart rate (>120/min)
2. Increased respiratory rate (>24/min)
3. Raised temperature (>38.5 °C)
4. Increased systolic blood pressure (>160 mmHg)
5. Increased muscle tone (Modified Ashworth Scale score)
6. Decerebrate or decorticate posturing
7. Profuse sweating

### Electronic data collection

Data collected will be stored in a database developed with the Research Electronic Data Capture system [64]. It is hosted on the server of the University of São Paulo. It is a software developed at Vanderbilt University (TN, USA). This is a fully Web-based software that enables electronic data collection and also study process management. It also respects the international policies on data privacy and security in the health sector [65].

### Blinding and allocation concealment committee

In the present study, there is a medical committee who can remove blinding, if something unexpected happens: a major adverse event, patient dropout, etc. The medical committee is not directly related to any patient group.

### Sample size calculation

The previously published studies about transcranial LED therapy are case reports or case series. Besides, there is no study which evaluated transcranial LED therapy for acute severe TBI patients. Considering these facts, the sample size was calculated based on the primary outcome proportion difference (favorable vs unfavorable) on chi-square with the estimation of effect size (Cohen’s *D*) of 0.5, alpha of 5%, and statistic power of 80%. The final result was 32 patients, who will be divided into two groups (16 patients each).

### Statistical analysis

The primary outcome variable will be analyzed with chi-square for comparison of dichotomized GOSE (favorable vs unfavorable) between intervention and sham. The second outcome measures for GOSE at 1 and 6 months after stimulation will be calculated in the same way as for the primary outcome measures. The results of the scales of DAI severity (Gennarelli’s), Adams, and Marshall will be compared with the results of the sham group with the Mann-Whitney *U* test. The second outcome measures related to hemodynamic evaluation will be analyzed with the *t* test for means and SD comparison of 1, 3, and 6 months with the basal value before stimulation.

The median and interquartile range will be calculated for age, intensive care unit days, hospitalization days, and days of treatment of infection. Gender, handedness, level of schooling, mechanism of injury, type of accident, sedation, pupils and presence of dysautonomia, presence of infection, and site of infection will be presented as absolute frequency, proportion, and confidence interval. The Glasgow Coma Scale score will be divided into four categories (3–5, 6–8, 9–12, 13–15) and will be expressed as absolute frequency, proportion, and confidence interval at four moments (scene, admission, discharged from ICU, and discharge from hospital).

Statistical Package for the Social Sciences software version 23.0 for Windows (Prentice Hall, Chicago, IL, USA) will be used for analyzing data. A significance level of *p* < 0.05 will be considered for data analysis.

### Ethical issues

This research involves a minimal risk to the patients, considering that transcranial LED stimulation is a neuromodulation technique tested in other studies [66–69] and with other proposals without severe adverse events reported.

## Discussion

This study protocol is designed to focus on investigating the neuromodulation role of transcranial LED therapy (TCLT) for patients who are victims of severe traumatic brain injury (TBI) with diffuse axonal injury. The objective of the study is to verify the hypothesis that this therapy presents pro-cognitive effects for rehabilitation.

Different studies have emerged to prove the positive effect of TCTL for neuropsychological diseases. After these results, some studies, most of which are case series [66–68], have approached the effects of this treatment for TBI with interesting results.

No specific clinical trials on intervention in the acute stage of TBI were found, which justifies the need for an investigation of the efficacy and effectiveness of TCLT. Considering the global impact of TBI on public health policies, the idea of a non-invasive therapy which helps in neurorehabilitation has an interesting appeal.

The first animal model described with TBI treated with TCLT (808-nm NIR/red LED) was performed with mice. Mice were submitted to only one TCLT section for 4 h. An important reduction (about 90%) of post-injury area size was noticed. Neurological severity scores were also significantly lower at 28 days for laser-treated mice [66]. A different approach with a 6-min daily TCLT session for 10 days reverted deficits in working memory tests of mice [67].

A two-case study with TCLT performed to improve cognitive function in chronic mild TBI patients found improvements in verbal learning, executive function, and memory [68]. With a higher number of patients with chronic mild TBI, the same author published positive results for 11 patients submitted to 18 outpatient sessions, starting at 10 months to 8 years post-TBI. In this study, each LED cluster head (5.35-cm diameter, 500 mW, 22.2 mW/cm^2^) was applied for 10 min to each 11 scalp placement (13 J/cm^2^). They evidenced improvements in executive function as well as sleep and fewer post-traumatic stress disorders [69].

Increasing the number of sessions, another series of two patients submitted to 18 red/NIR TCLT treatments (500 mW, 22.2 mW/cm^2^, 22.48 cm^2^ per treatment area) in chronic TBI patients (who began TCLT at 10 months to 8 years post-TBI) showed improvements in cognition (mainly executive function and verbal memory) [68].

Moderate TBI patients were treated with a high-power NIR laser (10–15 W at 810 and 980 nm) through 20 NIR applications over a 2-month period. They showed decreased headache, depression, anxiety, and insomnia, whereas cognition and quality of life improved, accompanied by changes in the SPECT imaging [70].

After 18 TCLT treatments (26 J/cm^2^ per LED cluster head placement red/NIR, 500 mW, 22.2 mW/cm^2^) in left-hemisphere stroke patients with chronic aphasia, resting-state fMRI scans have been obtained and shown significant increases in “naming ability” [10].

The 3-month period was chosen for the primary outcome because it presents an important relationship with the first year’s outcome. The usual result expected for severe TBI patients is about 30% of favorable outcome at 3 months [71]. Considering a strong size effect for the intervention, we expect a treatment effect of 50 percentage points (difference in the favorable-outcome rate of 30% vs 75%).

## Trial status

The registration of this trial on the Clinicaltrials.gov website was done on September 15, 2017. Recruitment started on October 10, 2017, when the helmets were available to start the research. The programmed completion date for the primary outcome is October 2018.

## Additional file

Additional file 1: SPIRIT 2013 checklist: recommended items to address in a clinical trial protocol and related documents. (DOC 123 kb)

## Abbreviations

ATP: Adenosine triphosphate
CCO: Cytochrome c oxidase
CONSORT: Consolidated Standards of Reporting Trials
CT: Computed tomography
DAI: Diffuse axonal injury
DTI: Diffusion tensor imaging
GCS: Glasgow Come Scale
GOSE: Glasgow Outcome Scale Extended
LED: Light-emitting diode
MRI: Magnetic resonance imaging
NIR: Near-infrared
NO: Nitric oxide
REDCap: Research Electronic Data Capture
SD: Standard deviation
SPIRIT: Standard Protocol Items: Recommendations for Interventional Trials
TBI: Traumatic brain injury
TCLT: Transcranial LED therapy
